# Detection of M-Sequences from Spike Sequence in Neuronal Networks

**DOI:** 10.1155/2012/862579

**Published:** 2012-07-18

**Authors:** Yoshi Nishitani, Chie Hosokawa, Yuko Mizuno-Matsumoto, Tomomitsu Miyoshi, Hajime Sawai, Shinichi Tamura

**Affiliations:** ^1^Graduate School of Medicine, Osaka University, Suita, Osaka 565-0871, Japan; ^2^Health Research Institute, National Institute of Advanced Industrial Scientific and Technology (AIST), Ikeda, Osaka 563-8577, Japan; ^3^Graduate School of Applied Informatics, University of Hyogo, Kobe, Hyogo 650-0044, Japan; ^4^Department of Integrative Physiology, Graduate School of Medicine, Osaka University, Suita, Osaka 565-0871, Japan; ^5^NBL Technovator Co., Ltd., 631 Shindachimakino, Sennan, Osaka 590-0522, Japan

## Abstract

In circuit theory, it is well known that a linear feedback shift register (LFSR) circuit generates pseudorandom bit sequences (PRBS), including an M-sequence with the maximum period of length. In this study, we tried to detect M-sequences known as a pseudorandom sequence generated by the LFSR circuit from time series patterns of stimulated action potentials. Stimulated action potentials were recorded from dissociated cultures of hippocampal neurons grown on a multielectrode array. We could find several M-sequences from a 3-stage LFSR circuit (M3). These results show the possibility of assembling LFSR circuits or its equivalent ones in a neuronal network. However, since the M3 pattern was composed of only four spike intervals, the possibility of an accidental detection was not zero. Then, we detected M-sequences from random spike sequences which were not generated from an LFSR circuit and compare the result with the number of M-sequences from the originally observed raster data. As a result, a significant difference was confirmed: a greater number of “0–1” reversed the 3-stage M-sequences occurred than would have accidentally be detected. This result suggests that some LFSR equivalent circuits are assembled in neuronal networks.

## 1. Introduction

The brain is recognized as a very large-scale network system in which the basic element is a neuron [1–4]. In recent studies of the memory mechanism in the brain, investigating a formation of information communication is more essential than specifying the region of memory in the brain [4].

The basic study of communication method in the brain is to clarify the coding mechanism of information. Therefore varieties of coding for neuronal information, for example, rate code, were proposed in previous studies [5–15]. The first theory of information architecture is cell-assembly theory proposed by Hebb in 1949 [16, 17]. Abeles postulated that “synfire chains” of spike with relatively fixed intervals could travel through the brain representing information and various behavioral states [18–21]. Rolston and others have observed a robust set of spontaneously repeating spatiotemporal patterns of neuronal activity using a template matching algorithm [22].

Then, the question arises as to how the data communication is controlled and what and how the form of controlled data communication is constructed. This question is essential to investigate the mechanism, how information is communicated in more detail. To resolve this question, decoding sequence pattern in one block of spike activity (analyzing time series patterns of firing), not a rate of spike or waveform of action potential, is necessary. However, in previous studies, the main discussion of information assemblies in neuronal network is propagation of firing rate or synchronization of firing timing in neuronal network in the broad view of spike activity; decoding sequence pattern such as described above has not been considered; therefore, there are few clues to understand the mechanism used in the brain for coding neuron spikes and communicating data.

**Figure 1:**
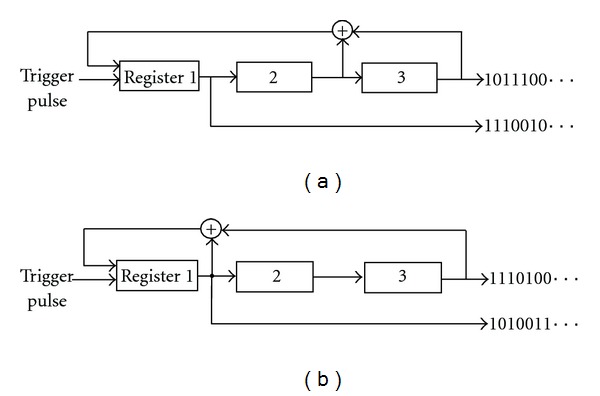
3-stage LFSR circuit, the operation at the ring sum is an exclusive-OR such as 1 + 0 = 1 and 1 + 1 = 0. (a) A basic circuit with feedback [2, 3] generates an M-sequence as 10111001011100…(basic pattern). (b) A mirror circuit of (a) with feedback [1, 3] generates a time course of reversed-order M-sequence, as 11101001110100….

 In circuit theory, a binary counter with *n*-bit logical elements (registers) can count up to 2^*n*^−1. With an adequate feedback link, the loop circuit becomes equivalent to a binary counter, the output of which becomes an M-sequence with length 2^*n*^−1 and is called the period; here, “M” stands for maximum length. If this resulting M-sequence is used as an intrinsic code of its own loop, 2^*n*^−1 loops can be discriminated [23]. For example, a 3-stage linear feedback shift register (LFSR) generates a 7-bits period M-sequence, as shown in Figure 1.

M-sequences perform most efficiently in synchronous communication and they are used for the control of data transmission, including code division multiple access (CDMA) for cell phones [23]. We assume that some LFSR circuits are assembled in neuronal networks to control data communications using M-sequences. Although this assumption has already been demonstrated by computer simulation in our previous study [24], physiological verification of this assumption has not been performed. Thus, the purpose of this study is to investigate LFSR circuits in neuronal networks in order to physiologically verify this assumption.

Cultured, small-scale neuronal networks on multielectrode arrays (MEAs) are feasible for analysis of network assemblies. MEAs can be used to apply stimulation pulse into neurons with sufficient flexibility and have been used to identify functional connections in neuronal networks [25–28].

 In this study, we investigate M-sequences from the time course of stimulated action potentials in neuronal networks grown on an MEA and discuss the LFSR circuit assemblies in neuronal networks from the detected M-sequence patterns.

## 2. Methods

### 2.1. Cell Cultures

Cell cultures of hippocampal neurons were dissected from Wistar rats on embryonic day 18. The procedure was performed in accordance with protocols approved by the Institutional Animal Care and Use Committee of AIST. Hippocampi were dissociated with 0.1% trypsin (Invitrogen, Tokyo, Japan) in Ca^2+^-free and Mg^2+^-free phosphate-buffered saline minus at 37°C for 15 min. The dissociated neurons were planted at a density of 3.3 × 10^5^ cells/mm^2^ in polyethyleneimine-coated MEA dishes (MED-P515A, Alpha MED Scientific, Kadoma, Osaka, Japan) with 8 × 8 planar microelectrodes. The size of each electrode was 50 × 50 *μ*m and the electrode spacing was 150 *μ*m. To locate neuronal networks in the central area of each MEA dish, we used a cloning ring with an inner diameter of 7 mm. The ring was removed the following day. Neurons adhered to the substrate of the MEAs covering all electrodes.

Neurons were maintained at 37°C in a humidified atmosphere that contained 5% CO_2_ and cultured for 21–40 days in Dulbecco's Modified Eagle's Medium (Invitrogen) that contained 5% horse serum and 5% fetal calf serum with supplements of 100 U/mL penicillin, 100 *μ*g/mL streptomycin, and 5 *μ*g/mL insulin. Half of the culture medium was renewed twice per week.

**Figure 2:**
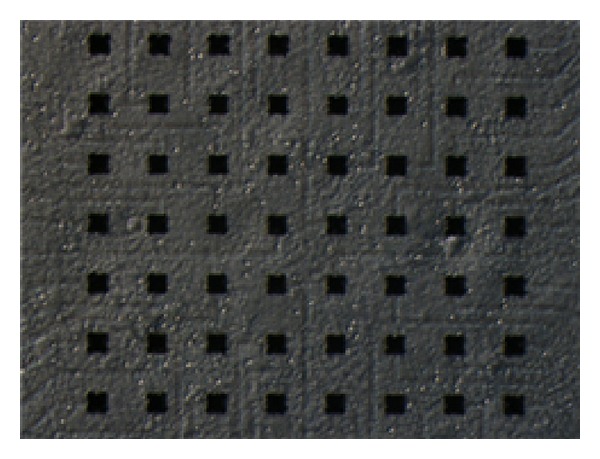
Micrograph of cultured hippocampal neurons in an MEA black rectangles are electrodes. The size of each electrode is 50 × 50 *μ*m and the electrode spacing was 150 *μ*m.

Figure 2 shows a micrograph of cultured neurons in an MEA.

In this study, we prepared 6 cultured cell samples at 22–50 days in vitro (DIV) and named them cultures 1–6.

### 2.2. Stimulated Spike Recording

 Stimulated spikes were recorded by an extracellular recording system with 64 channels (MED64, Alpha MED Scientific). The sampling rate of the recording was 20 kHz and the recording time was 3 s. Stimulation was applied at a particular channel (one electrode) 5 ms after the recording started. Stimulation was produced using a current-controlled bipolar pulse (positive, then negative) with a strength of 10 *μ*A and a duration of 100 *μ*s.

We tried template matching on some electrode on some cultures. Almost only one pattern of spike form was detected. Therefore, we did not do spike sorting [27] because there were few possibility that the action potentials originate from multiple neurons in our experiment.

### 2.3. M-Sequence Detection in Stimulated Spike Responses

The method we used to detect M-sequences in stimulated spike responses is as follows.

First, a raster plots were obtained by detecting peaks from recorded spike responses with a prespecified threshold on each channel [28] with a sampling frequency of 10 kHz.

Threshold was determined by trial and errors. In our experiments and most suitable threshold was 5 times of the RMS of noise (about 0.016~0.024 mV), which is able to reduce noise almost completely without reduceing action potential. This threshold was also reported as suitable in [28].

Then, the raster plot data was divided into the particular width of the time bins. The state of a bin was recognized as “1” if a spike existed; otherwise, it was recognized as “0.” If two spikes were detected in a bin, it was neglected. Then, the raster plot was converted to a time course of binary data in order to investigate sequence patterns.

Interval of spikes is dispread variously. So it is crucial to determine the bin size of M-sequences detection. Some problems of fixed bin always exist [29]. We consider that these problems were resolved practically by a statistical analysis on the number of detected M-sequences on multielectrode on multiculture as described in Chapter 4.

The maximum interval is about 30 ms in every culture. While, the minimum size of LFSR is 3 stage which generates a 7-bits (6 bins) period M-sequence [23] practically. Therefore, the maximum bin size of M-sequence is considered about 5 ms.

Considering various bin size of M-sequence as described above and sampling period of raster plot data (0.1 ms), time bins (discrete bit length of M-sequence) with multi-widths from 0.1 ms to 5 ms by increasing 0.1 ms step at a time were applied. Detection results on each time bin width were superimposed and plotted on a time axis. Then, it was able to detect various interval lengths of M-sequences though often with overlapping.

 Considering that the data communication must begin at state “1” because the start of communication could not be identified at state “0,” the detection of M-sequence patterns was started from state “1.”

The conversion of the data into a raster plot, converting the raster plot into a time course of binary data, and M-sequence detection were performed on a personal computer using detection programs implemented with MATLAB (MathWorks Japan, Tokyo, Japan).

## 3. Results

 Stimulated spike activities appeared in the duration of 100–300 ms and then completely disappeared after the duration of the time.

**Figure 3:**
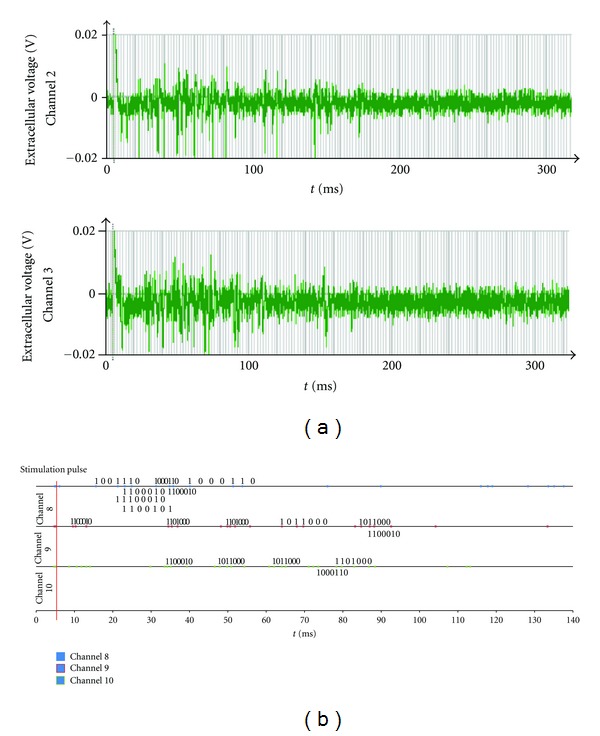
Stimulated spike response and results of M-sequence detection for culture 1. (a) The raw recording spike data (0-1 s). A stimulated spike response was observed from 5 ms (applied stimuli) to 150 ms on channels 2 and 3. (b) The raster plot and detected M-sequences on channels 8, 9, and 10.

A reverberation which seemed to be caused by potential energy of stimulation pulse was observed for 0.5 ms after stimulation.  Figure 3(a) shows the spike response on channels (ch) 2 and 3 for culture 1.  Table 1 shows the number of spikes evoked when the stimulation was activated and the time length when the stimulated spike response was observed for each culture.

50–55 channels, whose number of spike by the stimulation, was more than 20 with 100 ms after the stimulation were selected for analyzing M-sequence.

**Figure 4:**
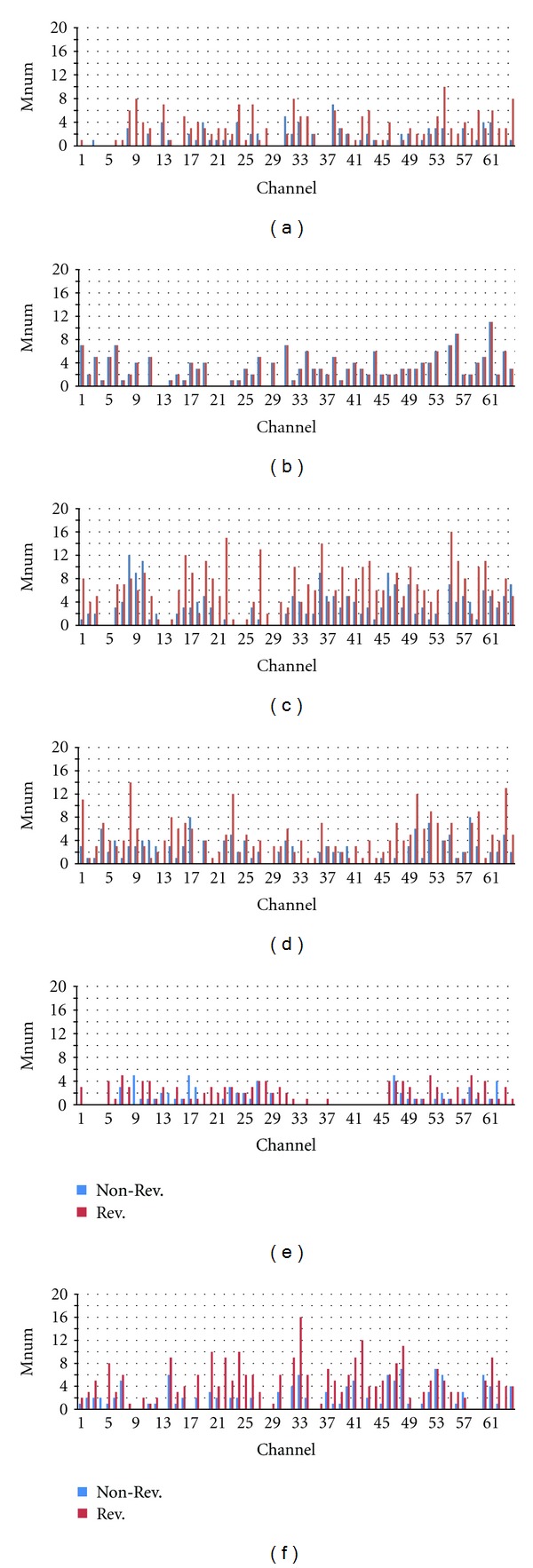
Number of detected M-sequences for (a) Culture 1, (b) Culture 2, (c) Culture 3, (d) Culture 4, (e) Culture 5, and (f) Culture 6.

We could detect several 3-stage M-sequences (M3), including those generated by mirror circuits and “0-1” reversed-state sequences (Rev. M3). Furthermore, although we attempted to find 4-stage M-sequences (M4) as 100110101111000…, we could not detect them in all the cultures that were part of this study.  Table 2 summarizes the M-sequence patterns detected from all the cultures in this study.  Figure 3(b) shows the result of the M-sequence detection on channels 8, 9, and 10 for culture 1. As shown in this figure, various patterns and interval lengths of M-sequences were detected. Based on the detection results, the total number of detected M-sequences (sum of all patterns) discriminating between non-Rev. M3 and Rev. M3 was counted for each channel, as shown in Figure 4.

Considering the possibility that some neurons might belong to plural circuits that could generate M-sequences simultaneously, we counted each sequence independently even if some sequence patterns were overlapped as shown in Figure 3(b).

## 4. Analysis and Discussions

 From the detection results, as previously described, some M-sequences were detected from the stimulated spike response. However, there is room for doubt whether this result shows the existence of LFSR circuits that generate M-sequence in neuronal networks. In this section, we provide a more detailed analysis on the detected sequence pattern and discuss the analysis in order to resolve these doubts.

### 4.1. Rate of the Number of Rev. M3 Patterns among Detected M-Sequences

 As shown in Table 2, 12 types of M-sequences were detected from the analysis results, among which 8 types were of non-Rev. M3 and 4 types were of Rev. M3 patterns.

Assuming that the sequence of non-Rev. M3 and Rev. M3 patterns shown in Table 2 are randomly generated, the probability of Rev. M3 pattern detection should be about 33.3%. However, from the analysis results, we noticed that the rate of the number of Rev. M3 patterns was significantly higher than this probability value, which was 73.4 ± 7.23% (the mean and standard division) for all cultures.

### 4.2. Significance of the Estimation of M-Sequence Detection Probability

Detected M3 patterns were relatively simple, constructed by only 4 interval patterns such as 11, 101, 1001, and 10001, and the number of spike intervals on each channel ranged from 20 to 40. Therefore, the possibility of an accidental detection of M-sequence patterns (without LFSR circuits generating M-sequences) is not zero. To resolve this doubt, we performed a hypothesis test to estimate the significant detection probability of M-sequences in observed stimulated spike sequences as follows.

**Figure 5:**
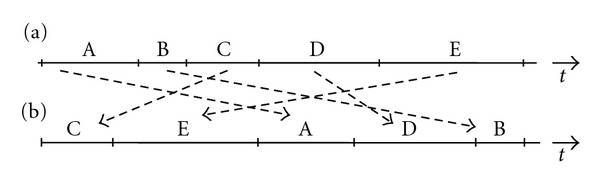
Process of interval shuffle (a) original raster plot data (b) shuffled raster plot data.

First, we generated a shuffled spike-interval sequence from the original observed raster data, called an interval shuffle, on each channel (Figure 5) [30]. The shuffled raster plot has the same number of state “1” and spike intervals as the original raster plot, but it can be considered to be a random sequence without LFSR.

Incidentally, there are some controversy about the shuffling method, using only one shuffling method is not enough to estimate the significant detection probability of M-sequences exactly [21, 31, 32]. However, most shuffling methods, for example, channel shuffling, spike shuffling across cells, spike exchange across cells [21], and so forth, involve breaking number of spike on each channel and spike interval, the confidence of the test result was lost except for the interval shuffle.

Therefore, in order to estimate the significant detection probability of M-sequences exactly, we performed the interval shuffle 20 times (created 20 shuffled interval data from an original spike data) instead of multiple shuffling methods. These shuffled interval data were considered the population as described below in detail.

Moreover, we also tested for some of the raster plots created from two random noise data; one of them was the raster plots obtained by detecting peaks from recorded noise responses of medium (without cell cultures) with threshold 0.01 (mV) and the other is a sequence data created from random numbers, to compare with raster data from stimulated spikes.

After the interval shuffle, the significant difference in the mean number of detected M3 patterns on one channel between the original observed raster data and interval shuffle data of each culture (population) was tested using a *z*-test on the assumption as follows.

- The total number of detected M3 on each channel is considered individual.
- The population is the group of individuals on the interval shuffle (20 times).
- The sample is the group of individuals on the original raster data.
- The standard deviation of the sample is equal to that of population.
- Both sample and population are normal distributions.

The null hypothesis H_0_ and alternative hypothesis H_1_ are as follows.

H_0_: The number of detected M-sequences from the original raster data is not larger than the number detected from the interval shuffle data.
H_1_: The number of detected M-sequences from the original raster data is larger than the number detected from the interval shuffle.

The mean number of detected M3 per channel in population *μ*_0_ is defined by the equation as follows:

(1) μ0=∑t=120{∑ch=1chnumt(mnumst,chchnumst)},

where *t* is the shuffled time, chnums_*t*_ is the number of channels detected M3 on shuffled time *t*, and mnums_*t*,ch_ is the number of detected M3 on shuffled time *t*, chnum ch.

The standard deviation of population *σ* is defined as

(2) σ=1∑t=120chnumst−1∑t−120{∑ch=1chnumst(mnumst,ch−μ0)2}.

Then, equation for the value of *z* is

(3) z=mnumo−−μ0σ/chnumo,

where $\overset{-}{\text{mnumo}}$ is the mean number of detected M3 per channel and chnumo is the number of channels that detected M3 on original data.

We tested by using the sum of Rev. M3 patterns and non-Rev. M3 patterns individually because we noticed a higher detection rate of Rev. M3 patterns, as previously described.

**Figure 6:**
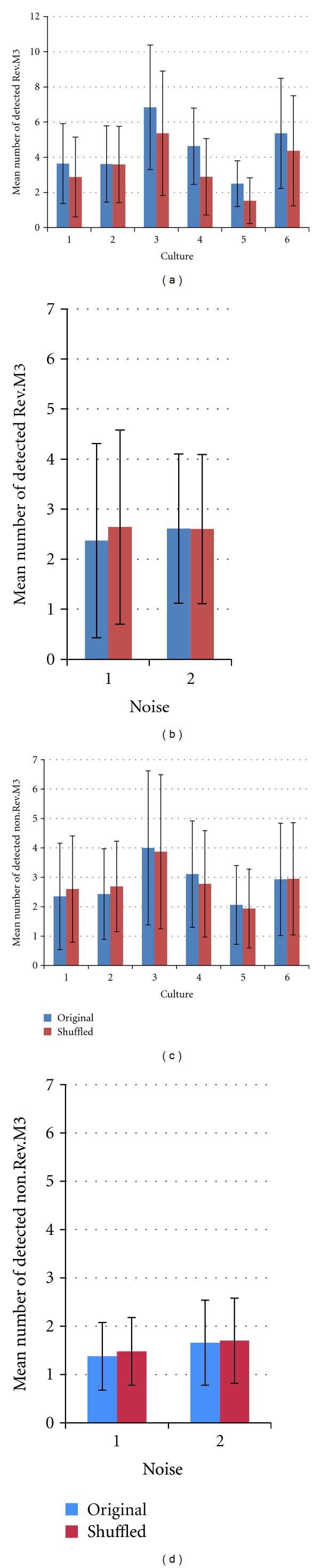
Mean number of detected M3 patterns. Error bar shows the standard deviation of the number of detected M3 patterns for each culture. (a) Rev. M3 from spike data. There were significant differences between the original and interval shuffle data in all cultures except culture 2 (*P* < 0.05). (b) Rev. M3 from random noise data. Noise 1 is the raster plots obtained by detecting peaks from recorded noise responses of medium (without cell cultures) with threshold 0.01[mV]. Noise 2 is random sequence data created from random numbers. (c) non-Rev. M3 from Spike data (d) non-Rev. M3 from Noise data.

From results of test, we confirmed that a significantly greater number of Rev. M3 patterns were detected from the original data than from the interval shuffle in all cultures except culture 2 as shown in Figures 6(a) and 6(c), while no significantly greater number of Rev. M3 patterns was detected in random noise data as shown in Figures 6(b) and 6(d) when we set *P* < 0.05 (estimated from *z* value). In cultures 1, 3, 4, and 5, significantly greater numbers were detected when we set *P* < 0.01 also.

### 4.3. Discussion of Analysis Results

 M-sequence patterns detected from interval shuffle were recognized as accidental detections, and they were not generated from LFSR circuits.

 Detected number of Rev. 3 was above chance in the stimulated spike activity, while detected number of Rev. 3 was not above chance in random noise.

By assessing these results, we determined that the detected Rev. M3s from the original raster plot of cultures were generated by some 3-stage LFSR circuits assembled equivalently in a neuronal network except culture 2.

In the meanwhile, the fact that detected Rev. M3s from random noise were not much above accident indicates no assembly of LFSR. Moreover, a few non-Rev. M3 patterns detected from the culture data are not generated by LFSR also because the number of detected non-Rev. M3 in original raster plot was not larger than in the interval shuffle data as shown in Figure 6(c).

Incidentally, the reason why Rev. M3, not non-Rev. M3, is generated especially is still unclear; we consider there is a possibility that a stable state, (transmitting/accepting less energy signals to/from other neurons) which has negative voltage of electric potential of neuron, is realized by state “0” and this fact causes the reversing “0” and “1.”

### 4.4. Model of Equivalent LFSR Circuit in Neuronal Network

To conclude the previous discussions, we recognize the existence of some 3-stage LFSR circuits that generate M3 patterns (especially Rev. M3 patterns) equivalently, which suggests the possibility that this phenomenon might be related to the data communication in neuronal networks. Abeles denoted that there are synchronizations of spike pattern in 3 neurons in “synfire chain theory” [18]. We consider the fact suggests that the reason of that 3-stage LFSR circuits, not 4-stage, 5-stage and so forth, are assembled mainly at least in the early stage of development of neuronal network.

Then, the question that neurons can function as a logical element (shift register and XOR shown in Figure 1) arises. Considering the fact that a neuron is able to become excited when the neuron has multiple connections with other neurons and spikes from these neurons arrived at simultaneously, it seems unlikely that one neuron corresponds to one element of LFSR (shift resister) because one-to-one connection of neuron pair is not effective to excite a neuron.

To resolve the contradiction as described above, we propose two types of equivalent 3-stage LFSR model in neuronal network as follows.

**Figure 7:**
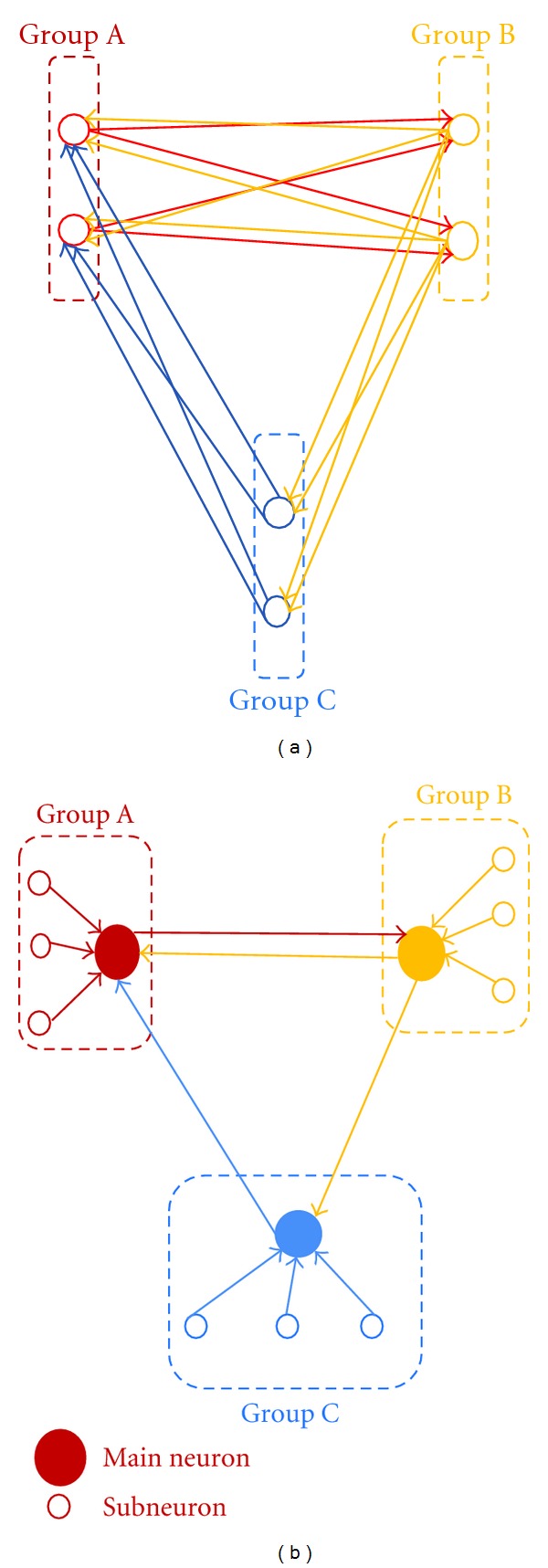
An equivalent 3-stage LFSR. (a) Model 1: spikes of multiple neurons in a neuron group propagate to another neuron group like synfire chain. Although we assume that neurons in the same neuron group evoke simultaneously and all pair of neurons are equal in their synaptic delay, there is a possibility that an equivalent LFSR circuit is assembled when the spike timing delay of each neuron in a same neuron group and synaptic delay between neuron groups are coordinated even if pair of neurons are not equal in their synaptic delay. (b) Model 2: the framework of LFSR is constructed by “*mainneurons”* and *“sub neurons”* are connected with “*mainneurons”* to excite them. Although, we omit a XOR function in both figures, some XOR circuit models constructed by neurons are already shown in previous study.

The first model is that spikes of multiple neurons in a neuron group propagate to another neuron group as shown in Figure 7(a). A neuron group corresponds to a shift resister. This propagation mechanism is similar to the theory of synfire chain [18–20]. We assume that neurons in the same neuron group evoke simultaneously and all pair of neurons are equal in their synaptic delay. Then synaptic delay corresponds to clock period of LFSR. From analysis results, the average time length of detected Rev. M3 is about 10 ms (Max. more than 30 ms, Min. 1 ms); therefore, the average clock period is about 1.67 ms (Max. more than 5 ms). These values do not contradict the value of synaptic delay (more than 1 ms) [31]. Collating the theory of synfire chain, we consider that these assumptions are proper. In the meanwhile, Izhikevich proposes a network model with a different synaptic delay [33]. We consider that there is a possible chance that an equivalent LFSR circuit is assembled when the spike timing delay of each neuron in a same neuron group and synaptic delay between neuron groups are coordinated even if pair of neurons are not equal in their synaptic delay.

The second model is that “*mainneurons”* which are connected with some *“sub neurons”* to excite *“mainneurons” *constructed LFSR as shown in Figure 7(b). This model also needs simultaneous spike timing of subneurons.

Then, we omit a XOR function in Figure 7, some XOR circuit models constructed by neurons are already shown in previous study [34].

Considering that patterns of network are an astronomically spread figure, there is a possibility that both types of circuit model as described above are assembled in neuronal network. There is a possibility that other types of models are assembled also. It is still unclear which type of model is appropriate.

## 5. Conclusion

We detected a significantly greater number of Rev. M3 patterns from the time series stimulated spike response than from the random series (interval shuffle) data in neuronal networks formed on MEAs. In conclusion, this result suggests that some equivalent 3-stage LFSR circuits are assembled in neuronal networks; detected M-sequences are generated by these circuits; they are not accidental potentials; and they are used for data communication in neuronal networks. We also proposed equivalent LFSR circuit in neuronal network.

Our future work will aim to identify the location and type of equivalent LFSR circuit, to resolve the reason why the major types of detected sequences are a Rev. M3 pattern by investigating data communications, to analyze the correlation between the culture term and the number of detected M-sequences to investigate growth process of equivalent LFSR, and to analyze the correlation between the scale of neuronal networks and the number of detected M-sequences.

Although we could find only M3 patterns in this study, there is a possibility that larger types of M-sequences can be detected in large-scale networks, for example, M4 and M5 patterns (generated on 5-stage LFSR circuits), which would be different from the small and early stages of cell cultures used as samples in this study.

Our studies suggest a new field of “computational brain architecture,” which can be applied to studies in brain physiology, brain machine interface, and related fields.

## Figures and Tables

**Table 1:** Average number of stimulated spikes on a channel (Num) and the time length for which stimulated spike response was observed (*L*).

	Culture 1	Culture 2	Culture 3	Culture 4	Culture 5	Culture 6
Num	26.9 ± 11.1	27.7 ± 9.47	45.7 ± 11.6	29.9 ± 10.7	17.8 ± 2.89	31.5 ± 17.9
*L*	160 ms	160 ms	240 ms	160 ms	75 ms	300 ms

**Table 2:** M-sequence patterns detected from all cultures. ^∗^Indicates generated by mirror circuit.

Type	Pattern
M3	1011100	
	1110010	
	1100101	
	1001011	
	1110100	^ ∗^
	1001110	^ ∗^
	1101001	^ ∗^
	1010011	^ ∗^

Rev. M3	1101000	
	1000110	
	1011000	^ ∗^
	1100010	^ ∗^
